# Synthesizing of Novel Bulk (Zr_67_Cu_33_)_100−*x*_W*_x_*(*x*; 5–30 at%) Glassy Alloys by Spark Plasma Sintering of Mechanically Alloyed Powders

**DOI:** 10.3390/molecules25081906

**Published:** 2020-04-20

**Authors:** M. Sherif El-Eskandarany, Naser Ali

**Affiliations:** Nanotechnology and Applications Program, Energy and Building Research Center, Kuwait Institute for Scientific Research, Safat 13109, Kuwait; nmali@kisr.edu.kw

**Keywords:** solid-state reaction, glass forming ability, metastable phase, powder consolidation, thermal stability, microhardness

## Abstract

Metallic glassy alloys with their short-range order have received considerable attention since their discovery in 1960’s. The worldwide interest in metallic glassy alloys is attributed to their unique mechanical, physical, and chemical properties, which cannot be found together in long-range order alloys of the same compositions. Traditional preparation methods of metallic glasses, such as rapid solidification of melts, always restrict the formation of glassy alloys with large atomic fraction (above 3–5 at%) of high melting point metals (Ta, Mo, W). In this study, (Zr_67_Cu_33_)_100−*x*_W*_x_*(*x*; 5–30 at%) metallic glassy alloys were fabricated through a mechanical alloying approach, which starts from the elemental powders. This system shows excellent glass forming ability in a wide range of W (0 ≤ *x* ≥ 30 at%). We have proposed a spark plasma sintering technique to prepare nearly full-dense large sized (20 × 20 mm) bulk metallic glassy alloys. The as-consolidated bulk metallic glassy alloys were seen to possess high thermal stability when compared with the other metallic glassy systems. This is implied by their high glass transition temperature (722–735 K), wide range of supercooled liquid region (39 K to over 100 K), and high values of crystallization temperature (761 K to 823 K). In addition, the fabricated ternary systems have revealed high microhardness values.

## 1. Introduction

Metallic glassy alloys, which are solid materials that consist of metallic atoms arranged in a random manner with no obvious long-range order fashion, have seen enormous development during recent years [1]. The world of materials science included this type of promising materials among the high-tech advanced materials since 1960, following the pioneering discovery by Duwez and coworkers [2]. With reference to their unique physical, chemical, and mechanical properties [3], bulk metallic glasses (BMG) have attracted numerous number of researchers of different schools [4,5,6,7]. BMG possesses desirable properties that are quite different from the corresponding long-range order materials with the same composition [8,9,10,11,12,13,14,15]. The term glass is nowadays almost unanimously used for an amorphous substance, which can be obtained through different ways of preparations, in particularly rapid solidification from the melts [2].

Metallic glassy alloys can combine different attractive properties, such as excellent mechanical ductility and formability in the supercooled liquid region, and yield strength [16,17,18], high magnetic permeability [19,20], low coercive forces [21], unusual corrosion resistance [22], and temperature-independent electrical conductivity [23]. Unluckily, the melt spinning process, conducted by rapid cooling rate that reaches to 10^6^ K/s of molten metal alloy, cannot be employed for those systems revealing a significant gap difference in the melting points between their alloying elements. In addition, it is difficult to use the rapid solidification approach for systems have low negative values of enthalpy change of formation (∆H^For^).

In contrast to the rapid solidification approach, Koch et al. reported the first novel technique for formation of Ni_60_Nb_40_ amorphous alloy by the mechanical alloying (MA) technique, using a high-energy ball milling of elemental Ni and Nb powders [24]. In this process, the solid-state reaction between two (or more) elemental metal powders (diffusion couples) is conducted at an ambient-temperature under inert gas atmosphere. The simplicity of the MA method and its capability of preparing very difficult systems led to the formation of large number of amorphous and metallic glassy alloys that cannot be obtained by the conventional rapid solidification method, such as Al-Ta [25], Al-Nb [26], Al-Zr [27], and Fe-W [28]. Zirconium (Zr)- based glassy alloys are well known glassy-forming alloys that can be prepared with a wide composition range (examples are given in Refs. [29,30,31,32]). In order to enable Zr-based metallic glassy systems for high temperature applications, their crystallization temperatures need to be increased. This will ensure their thermal stability during working operation conditions at temperatures below their crystallizations. In addition, the supercooled liquid regions (∆T_x_), which denote the difference between the crystallization (T_x_) and glass transition (T_g_) temperatures, should be also increased. Significant atomic fractions of refractory alloying, such as Ta, Mo, and/or W metals, can be added to enhance the glass forming ability (GFA) and to improve the crystallizations behavior of Zr-based metallic glasses. The selected alloying elements need to show large differences in sizes in order to form a complex structure that can withstand crystallization [33]. Technically, it is very difficult to prepare a homogeneous bulk glassy system, which contains large atomic fractions (<3–5%) of refractory metals by the conventional melting, casting, and rapid solidification techniques [34].

The present work has been addressed in order to investigate the effect of adding W metal in the range of 5–30 at% on the GFA and crystallization behavior of metallic glassy Zr_67_Cu_33_ alloy powders. For the purpose of this work, the cold rolling (CR) and MA approach, using high energy ball milling (BM) techniques, were employed to fabricate the desired metallic glassy systems with different W concentrations. The end-product of the fabricated glassy powders were then consolidated into BMG, while using the spark plasma sintering (SPS) approach. Due to its individual spark discharge effect, leading to unique shrinkage behaviors and densification, SPS has become a very attractive technique for preparing BMG with large size [35,36]. With reference to the aim of the present study, five individual (Zr_67_Cu_33_)_100−*x*_W*_x_* were prepared under the same experimental conditions and characterized with different techniques. Table 1 lists the chemical analysis obtained by inductively coupled plasma (ICP) mass spectrometry for the nominal- and final- compositions.

## 2. Results and Discussion

### 2.1. Morphological and Structural Changes with Changing the CR and BM Times 

**Figure 1:**
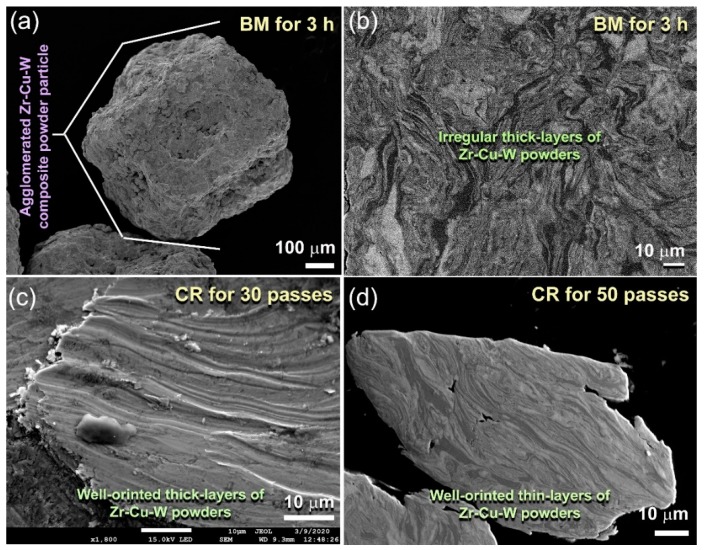
(**a**) Field-emission high-resolution transmission electron microscope (FE-SEM) micrographs of (Zr_67_Cu_33_)_70_W_30_ obtained after ball milling (BM) for 3 h. The cross-sectional view of the powder particle presented in (**a**) is shown in (**b**). The cross-sectional view of the powders milled for 3 h and then cold rolled (CR) for 30 and 50 passes are displayed in (**c**) and (**d**), respectively.

Field-emission scanning electron microscope (FE-SEM), X-ray diffraction (XRD), and field-emission high-resolution transmission electron microscope (FE-HRTEM) techniques were dedicated to follow the morphological and structural changes of (Zr_67_Cu_33_)_100−*x*_W*_x_* powders upon CR and then BM for different processing stages, taking the (Zr_67_Cu_33_)_80_W_30_ system as a typical example. The starting feedstock powders were firstly ball milled with low energy ball mill for 3 h to ensure a good mixture. After this stage, the powders were agglomerated to form larger particles (>850 mm in diameter), as displayed in Figure 1a. The SEM micrograph of the polished powder’s surface revealed multilayered structure with a random layer-orientation corresponding to the alloying elements, as shown in Figure 1b.

The as-BM powder obtained after 3 h of milling was charged into stainless steel tube and sealed under argon atmosphere inside a glove box in order to develop uniform layered-structure morphology with better orientation. The tubes containing the powders were then severely subjected to continuous CR for 30 passes. The CR powders were experienced from plastic deformations, leading to a reduction in their entire layer thickness and improvement to their orientation, as presented in Figure 1c. Further CR passes (50) were necessary to obtain ultrathin intimate layers in order to reduce the thickness of the metallic layers (6 μm ≤ layer thickness ≤ 1 μm), as displayed in Figure 1d.

**Figure 2:**
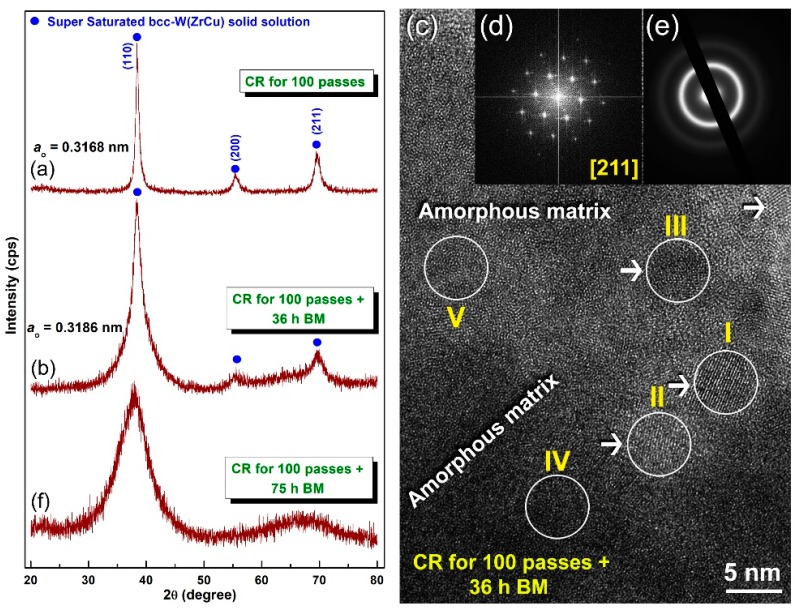
X-ray diffraction (XRD) patterns of (Zr_67_Cu_33_)_70_W_30_ powders obtained after (**a**) CR for 100 passes, and (**b**) CR for 100 passes and then 36 h BM. The high resolution transmission electron microscopy (HRTEM) image of the sample obtained after CR for 100 h followed by 36 h BM is displayed in (**c**) together with the corresponding nano beam diffraction patters taken from zones I (**d**) and II (**e**). The XRD pattern of the powders processed for 100 CR passes followed by BM of 75 h is displayed in (**f**). The elemental analysis conducted by local energy-dispersive X-ray spectroscopy (EDS) indicated a composition similarity in the indexed zones (I to V).

**Figure 3:**
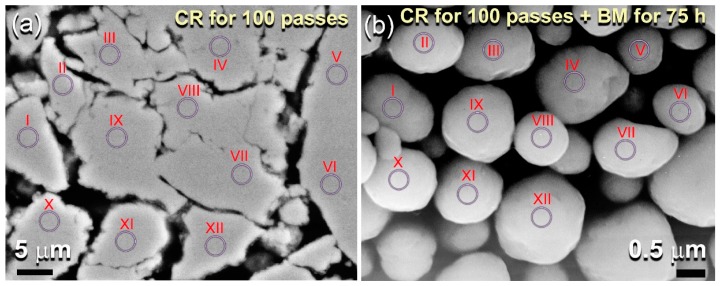
(**a**) FE-SEM micrographs of the cross-sectional view for ball milled (Zr_70_Cu_20_Ni_10_)_80_W_20_ powders obtained after 12.5 h of mechanical alloying (MA) time. The high-magnification micrograph of the powders obtained after the final stage of MA (100 h) is displayed in (**b**) Zones.

Figure 2a presents the XRD pattern of the powders obtained after 100 passes of CR, where the corresponding cross-sectional SEM micrograph of the polished surface is shown in Figure 3a. The layered-like microstructure morphology has surprisingly disappeared (Figure 3a) and, therefore, indicates the completion of CR-induced solid-state reaction and formation of a new phase. The XRD analysis of this sample (Figure 2a) confirmed the formation of a new reacted phase, as suggested by the presence of single domain (Figure 2a). Furthermore, the structural analysis indicates that the powder CR for 100 passes revealed a crystal structure corresponding to bcc-W metal, as indexed by the (110), (200), and (211) Bragg-peaks (Figure 2a). The lattice that the parameter (a_o_) calculated from the major peak (110) of this sample was 0.3168 nm. This value, which is larger than that one reported for pure W metal (0.3165 nm (PDF # 04-806)), indicates a solid-state diffusion of the alloying elements (Zr and Cu) into the W lattice to form solid solution metastable phase.

It can be concluded that the CR technique led to conducting a solid-state reaction of the diffusion couples of elemental Zr, Cu, and W metallic powder to obtain supersaturated bcc-solid solution phase without the existence of unprocessed crystals related to the starting feedstock metal powders. The same results were obtained for the other compositions contained lower concentration of W metal (>30 at%). We should emphasize that further CR passes (200 passes) did not lead to any phase transformations, where the product remained similar to the one obtained after CR for 100 passes.

The as-obtained bcc-W(ZrCu) solid solution possessed uniform elemental distribution with outstanding homogeneity in composition, as confirmed by local energy-dispersive X-ray spectroscopy (EDS) examinations for different 12 zones that are indexed in Figure 3a. The analysis, which is presented in Table 2a, indicates the formation of a homogeneous phase without severe compositional fluctuation or degradation, as characterized by the closed elemental composition of each zone with the starting nominal composition shown in Table 1.

The cold rolled powders were discharged from the stainless steel tube and charged in tool steel vial together with 50 tool steel balls in helium atmosphere glove box in order to understand the effect of high-energy ball milling on the crystal structure of the solid solution powders obtained after 100 passes of CR. The system was then BM for 36 h at ambient temperature, while using planetary ball mill. Figure 2b displays the XRD pattern of the powder obtained after 36 h of BM. The Bragg peaks related to W(ZrCu) solid solution tended to shift to the low angle side, suggesting an increase in the value of a_O_ (0.3186 nm), as presented in Figure 2b. It is worth notifying that the Bragg peaks of this solid-solution phase obtained after 36 h of BM revealed outstanding broadening (Figure 2b), either due to internal stresses and defects and grain refining achieved by BM (Figure 2b), or due to the existence of an amorphous phase. The HRTEM technique was employed to realize to understand the local structure of the powders obtained after 36 h and to realize whether the metastable bcc-W(ZrCu) solid solution preferred to transform into another metastable phase (amorphous) upon BM process or not.

The HRTEM micrograph of the powders that were obtained after 36 h of BM (Figure 2c) revealed rather heterogeneous structure contained nanograins of bcc-W(ZrCu) solid-solution (zones I, II, and III), together with featureless maze structure of an amorphous phase (zones IV and V), as shown in Figure 2c. The nano beam diffraction patterns (NBDPs) taken from zone 1 and zone V confirmed the existence of the bcc-solid solution phase (Figure 2d) that overlapped with an amorphous phase (Figure 2e). It can be then concluded that, when the powders experienced severe plastic deformation generated by the ball-powder-ball collision, the solid-solution tended to transform into a less stable phase (amorphous). It is worth mentioning here that the elemental composition investigated by the EDS technique of the zones shown in Figure 2c were very closed to each other. This implies that the solid-solution-to-amorphous W(ZrCu) phase transformation was conducted without compositional changes. The XRD pattern of the end-product that was obtained after 100 h of BM revealed a broad halo peak with the absence of nanocrystalline phase related to W-solid solution, as elucidated in Figure 2f. Since the ball milling technique is an energetic process in which the metastable materials tends to gain more energy upon introducing lattice imperfections (e.g., point defects and dislocations) [9], we claimed that W-solid solution phase transformed from a less stable phase to a more stable phase (amorphous) upon increasing the BM time. Towards the end of BM processing time (100 h), the as-obtained amorphous powders possessed excellent morphological characteristics, as indexed by their spherical-like morphology and narrow size distribution, as displayed in Figure 3b. Moreover, they revealed excellent elemental distributions beyond the micro level, as indicated by the near composition for each individual powder particles (Table 2b).

**Figure 4:**
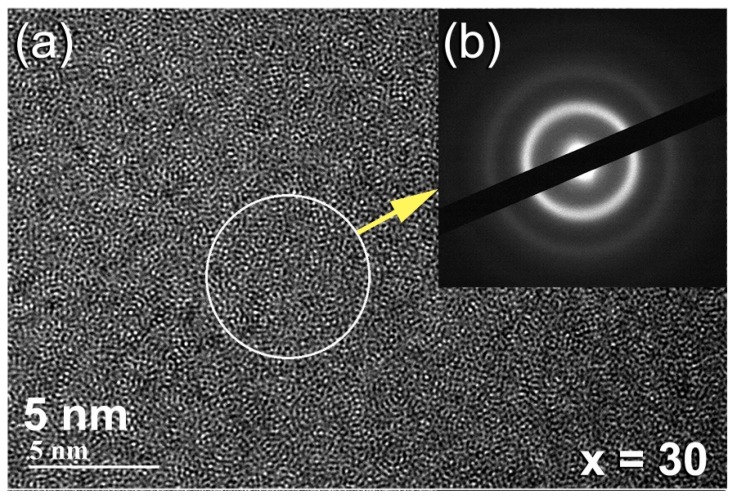
The FE-HRTEM micrograph and corresponding nano beam diffraction pattern (NBDP) of as-CR (Zr_67_Cu_33_)_70_W_30_ for 100 passes and then BM for 75 h are displayed in (**a**) and (**b**), respectively.

The HRTEM image of the powders that were obtained after 100 h of BM is presented in Figure 4a, together with the corresponding NBDP (Figure 4b). The sample appeared featureless, revealing maze contrast of amorphous structure, with no indication of precipitations of any crystalline phases that were related to bcc-W(ZrCu) solid solution (Figure 4a). Moreover, the NBDP displays typical spot-free halo-diffraction of amorphous phase, which was clearly seen (Figure 4b).

**Figure 5:**
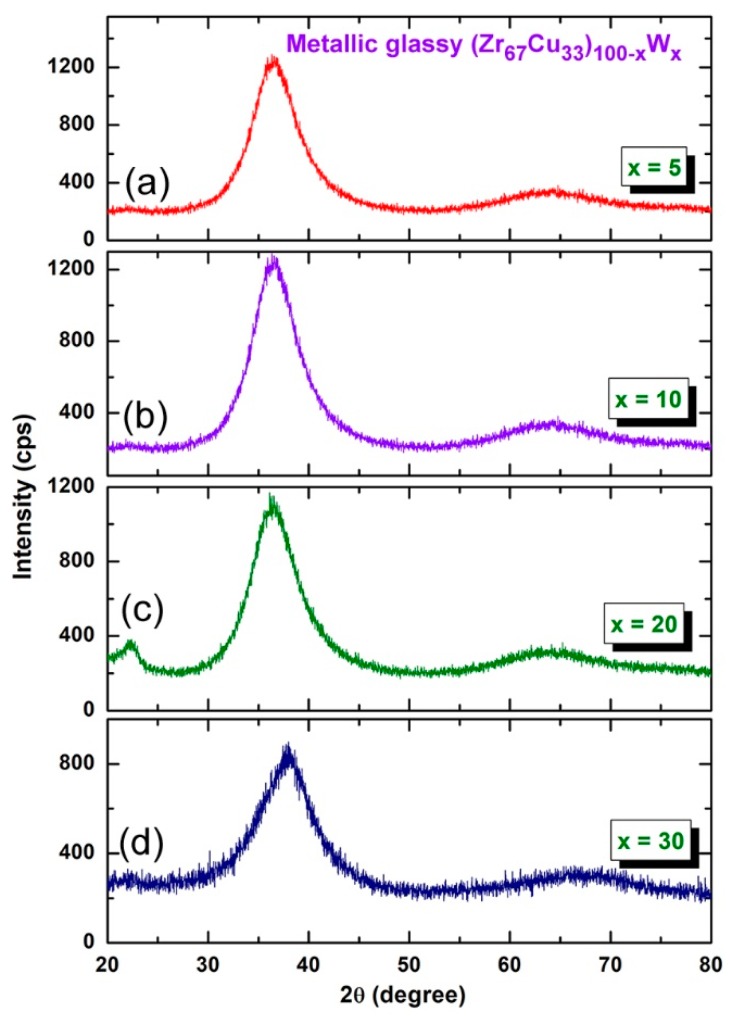
XRD patterns of (Zr_67_Cu_33_)_100−*x*_W*_x_* powders with *x* equals to (**a**) 5, (**b**) 10, (**c**) 20, and (**d**) 30 at%. The samples were obtained after CR for 100 passes followed by 75 h of BM.

Figure 5 presents the XRD patterns of the final-product of (Zr_67_Cu_33_)_100−*x*_W*_x_* (*x*; 5, 10, 20, and 30 at%) powders obtained after CR for 100 passes + BM for 75 h. The system has shown excellent GFA, as implied by the formation of halo-diffuse amorphous patterns in all W-range without overlapping with unprocessed crystalline phase (s), corresponding to solid-solution metastable phase (Figure 5a–d).

### 2.2. Thermal Stability

**Figure 6:**
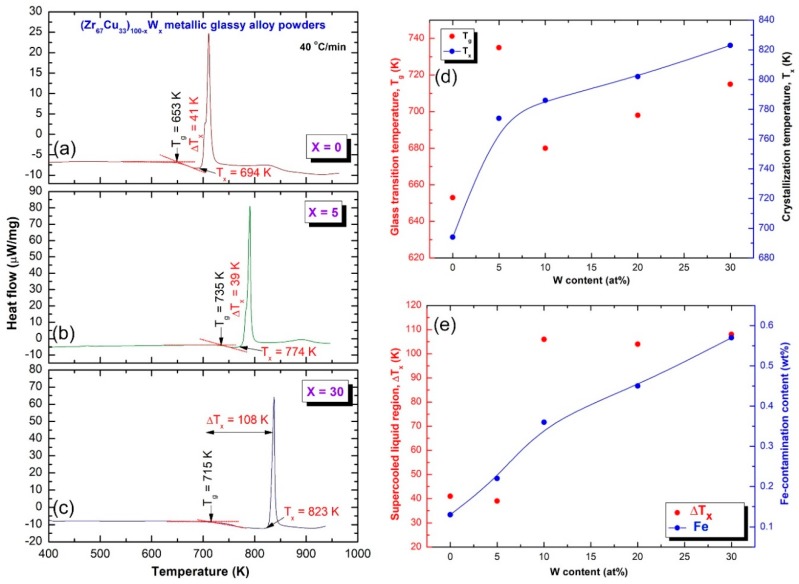
Differential scanning calorimeter (DSC) thermograms of CR (Zr_67_Cu_33_)_100−*x*_W*_x_* for 100 passes followed by BM for 75 h with *x* equaled to (**a**) 0, (**b**) 5, and (**c**) 30 at%. Dependence of T_g_ and T_x_ on W content is displayed in (**d**), where the effect of W content on ∆T_x_ and Fe-contamination content is elucidated in (**e**).

The differential scanning calorimetry (DSC) technique was employed to investigate the crystallization behavior of amorphous (Zr_67_Cu_33_)_100−*x*_W*_x_* powders. Figure 6a,c display the DSC thermograms of the prepared samples *x*; 0 to 30 at%. All of the samples revealed two opposite thermal events taken place at different temperatures. The first events were endothermic reactions achieved at lower temperatures in the range of 653 to 735 K, as presented in Figure 6. These endothermic reaction peaks are related to the glass transition (T_g_) of metallic glassy (Zr_67_Cu_33_)_100−*x*_W*_x_* phases, in which the metallic solid-amorphous transformed into liquid-amorphous (metallic glass) without structural changes [37]. The second events were characterized by the sharp pronounced exothermic peaks, which took place at the higher temperatures (T_x_) of 830 K, as presented in Figure 6. These exothermic reactions have resulted due to the crystallization process of the metallic glass where the amorphous short-range order transformed into a long-range order structure. The regions that are extended between the endothermic-and exothermic- reactions are referred to the supercooled liquid region (∆T_x_ = T_x_ − T_g_).

The onset T_x_ is usually used to characterize the thermal stability of the glass alloy, whereas the ΔT_x_ is used to characterize the GFA [18]. The (Zr_67_Cu_33_)_100−*x*_W*_x_* metallic glassy powders possessed high T_g_ in the range between 653 K to 735 K, as shown in Figure 6a–d. This system clearly showed that the ΔT_x_ has become wider upon increasing the W concentration, in the range between 41 K to 108 K, as presented In Figure 6e. Moreover, the (Zr_67_Cu_33_)_100−*x*_W*_x_* metallic glassy system showed high values of T_x_, which tends to increase with the increase in W content, as displayed in Figure 6b–d. The wide values of ΔT_x_ and high T_x_ imply the formation of metallic glassy phase with good GFA and high thermal stability. Increasing the mole fraction of the hard W powders led to introducing rather high Fe-contamination that came upon using steel milling tools. The Fe mole fraction was monotonically increased from about 0.15 wt% (x = 0) to reach to 0.58 wt% in the W rich side (30 at%). The existence of high temperature Fe phase might have had a marginal role in increasing the T_x_, as well as decreasing ∆T_x_. More studies related to this issue should be attained.

### 2.3. Fabrication of Bulk Metallic-Glassy (Z_67_Cu_33_)_100−x_W_x_ Alloys by SPS Technique

A spark plasma sintering technique (SPS) was used to consolidate the powders obtained after 75 h of BM to obtain more useful physical and mechanical information about this newly introduced metallic glassy systems fabrication approached. With reference to the W concentration of each system, the SPS consolidation step was achieved at temperature that ranged from 665 K to 780 K. This temperature range, which laid into the ∆T_x_ region, being above the T_g_ and far below T_x_ for all metallic glassy (Z_67_Cu_33_)_100−*x*_W*_x_* alloy systems, as shown in Figure 6. For all compositions, the consolidation procedure was conducted under vacuum with an axial load of 10 MPa. In the SPS process, the die and powder were both directly heated by the Joule effect of the direct current (DC), as described elsewhere [38].

**Figure 7:**
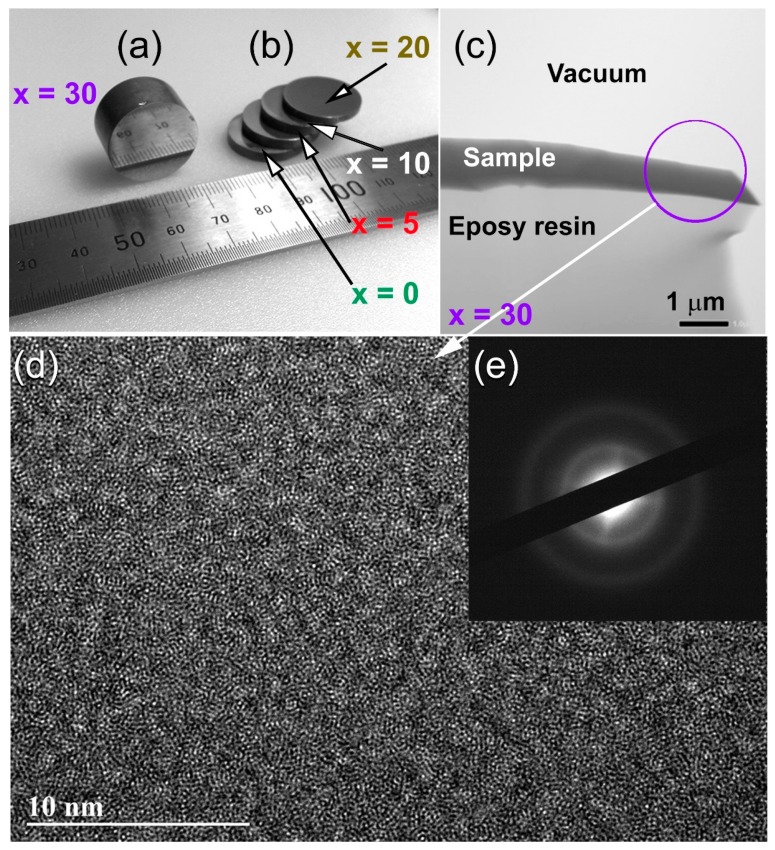
(**a**,**b**) A photo shows the outer morphology of Spark Plasma Sintering (SPS) (Zr_70_Cu_20_Ni_10_)_100−*x*_W*_x_* BMG consolidated buttons with diameter to height aspect ratios of 1:1 (**a**) and 0.5:1 (**b**). A low-magnification TEM micrograph of sliced sample prepared by cryo ion slicer approach is displayed in (**c**). The sample was mountained on epoxy resin supporter and fixed on single-tilt TEM-sample holder. The FE-HRTEM image of the zone indexed in (**c**) is displayed in (**d**) together with its corresponding NBDP (**e**).

Figure 7a displays the outer shape of as- SPS (Zr_67_Cu_33_)_70_W_30_ cylindrical buttons. Obviously, the consolidated object was revealed to be dense and smoothed surface, and it possessed metallic luster without any indications of the existencing cracks or pores, as presented in Figure 7a.

The button samples were sliced into ultrathin strips of less than 90 nm, using cryo ion slicer, in order to ensure the homogeneity of the as-consolidated button and the absence of any undesired phases that might be formed as a result of crystallizations SPS step. The thin strips were then fixed on epoxy resin before being mounted on a TEM sample holder. Figure 7c displays the low-magnification TEM micrograph of the elevation view for (Zr_67_Cu_33_)_70_W_30_ BMG sliced sample mountained on epoxy resin supporter. The FE-HRTEM micrograph that was taken from the indexed zone area shown in Figure 7c is displayed in Figure 7d, together with its corresponding NBDP (Figure 7e). Obviously, the consolidated sample reveled featureless maze structure with short-range order atomic distributions (Figure 7). Moreover, no indication of precipitations of any crystalline phases could be detected, where the NBDP displayed a typical halo-diffraction pattern of an amorphous phase (Figure 7e). Based on HRTEM investigation, it can be claimed that the SPS consolidation step can be successfully employed to prepare BMG alloys with large dimensions, starting from the milled powders.

**Figure 8:**
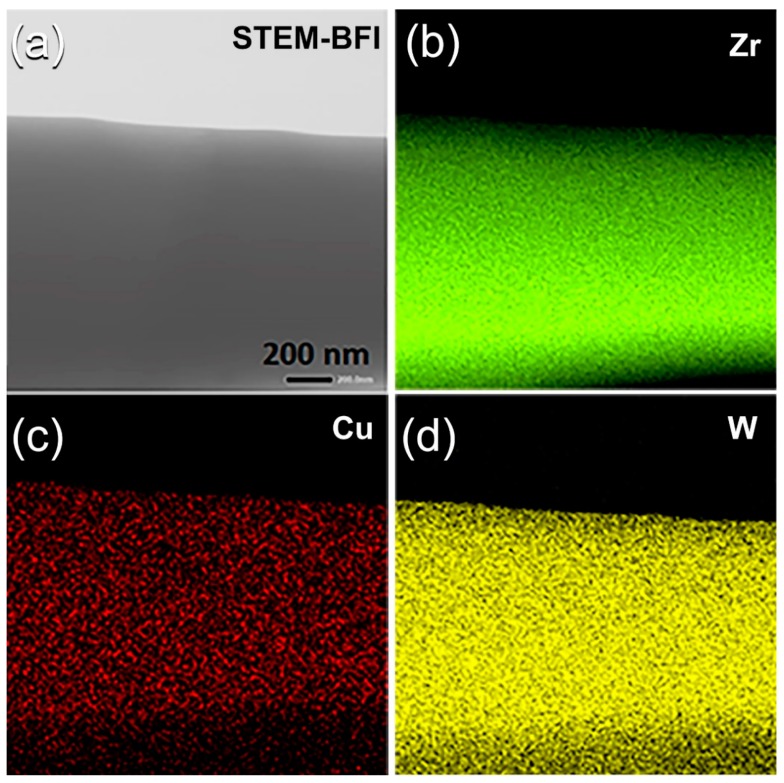
(**a**) Scanning transmission electron microscope-bright field image (STEM-BFI) and corresponding X-ray EDS elemental mapping of (**b**) Zr, (**c**) Cu, and (**d**) W for a sliced (Zr_67_Cu_33_)_70_W_30_ BMG sample.

Scanning transmission electron microscope (STEM), together with EDS analysis, were conducted to understand the elemental distribution beyond nanolevel of consolidated BMG buttons. Figure 8a presents the STEM of the bright field image (BFI) or the plan view of SPS (Zr_67_Cu_33_)_70_W_30_ consolidated BMG sample. The image, which possessed fine morphology, indicated the formation of non-crystalline fine structure with the absence of crystalline grains in good agreement with the FE-HRTEM image that were taken for the same sample (Figure 7d).

The X-ray EDS elemental maps that were taken for the alloying elements of Zr (Figure 8b), Cu (Figure 8c), and W (Figure 8d) were excellently distributed without any indications of the undesired compositional gradient of fluctuations. The average chemical composition, which was taken from 10 individual observations of this sample, was 37.94 wt%-Zr, 13.08wt%-Cu, and 48.98 wt% W is being very closed to the nominal composition that is shown in Table 1. This implies that the SPS procedure can be achieved without compositional degradation [38,39,40].

**Figure 9:**
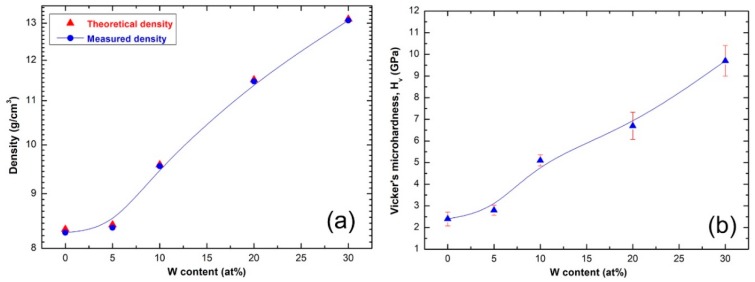
(**a**) Bulk density and (**b**) Vickers microhardness measure for SPS (Zr_67_Cu_33_)_100−*x*_W*_x_* BMG consolidated system.

Figure 9a summarizes the results of the densities measured (circular symbols) for SPS (Zr_67_Cu_33_)_100−*x*_W*_x_* consolidated BMG samples. Each point in the graph was obtained from the average of three individual measurements, using Archimedes’ principle. The results were compared with the theoretical densities that were calculated from the mixing principle and plotted in Figure 9a (triangle symbols). Figure 9b presents the correlation between W content and the measured Vickers microhardness for SPS (Zr_67_Cu_33_)_100−*x*_W*_x_* consolidated BMG samples. The measured density for binary BMG Zr_67_Cu_33_ system (8.276 g/cm^3^) was monotonically increased with increasing W content from 5 to 10 at% to be 8.366 and 9.55 g/cm^3^, respectively, as displayed in Figure 9a. A drastic increasing in the density values was realized upon further increase in W to 20 (11.468 g/cm^3^) and 30 (13.081 g/cm^3^) at%, as shown in Figure 9a. This nearly linear increase trend in the density is attributed to the monotonic increase of high-density W alloying element (19.35 g/cm^3^) in the (Zr_67_Cu_33_)_100−*x*_W*_x_* BMG system.

The reported Vickers microhardness (H_v_) values of pure alloying elements of the present BMG are 0.343–0.369 GPa (Cu), 0.820–1.8 GPa (Zr), and 3.43–4.6 GPa (W) [41,42,43,44]. The influence of W additive on the measured Hv for (Zr_67_Cu_33_)_100−*x*_W*_x_* system, in the range between W; 5 at% to 30 at%, is shown in Figure 9b. The Hv−W content show a neatly linear relationship, in which H_v_ slightly increased from 2.4 ± 0.32 GPa for binary BMG Zr_67_Cu_33_ system to 2.8 ± 0.23 GPa upon adding 5 at% W (Figure 9b). This value was jumbled to 5.1 ± 0.25 GPa and 6.7 ± 0.63 GPa upon the increase W to 10 at% and 20 at%, respectively, as displayed in Figure 9b. The SPS (Zr_67_Cu_33_)_70_W_30_ consolidated BMG sample shows an extraordinary high Hv value for any BMG system, which is measured and reported to be 9.7 ± 0.71 GPa (Figure 9b).

## 3. Materials and Methods

**Figure 10:**
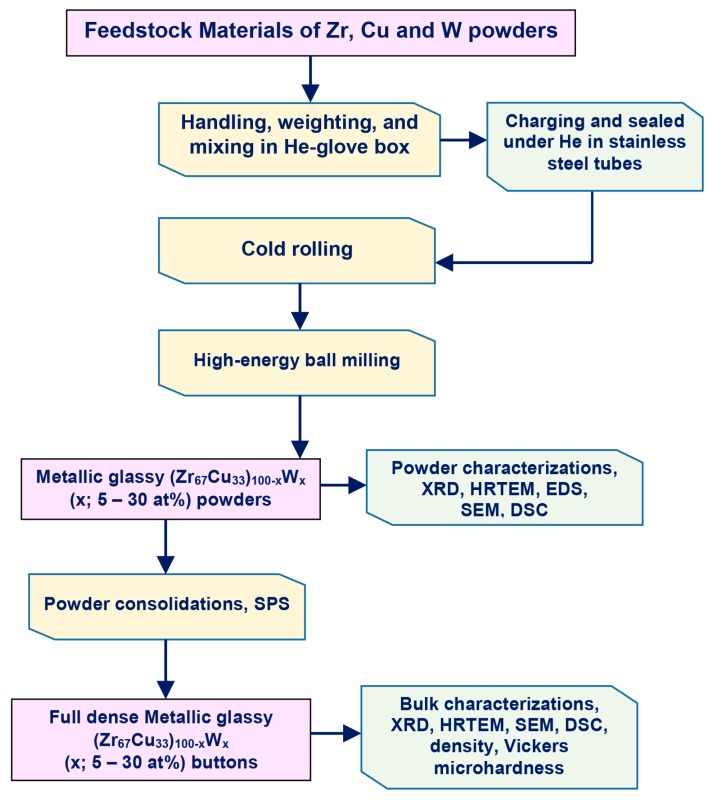
Flowsheet diagram presenting the experimental procedure followed in the present study for preparations and characterizations of metallic glassy (Zr_67_Cu_33_)_100−*x*_W*_x_* materials.

Figure 10 displays a schematic flowsheet diagram, which elucidated the experimental procedures used in this study for the preparations and characterizations of metallic glassy materials.

### 3.1. Feedstock Materials

Pure elemental powders of metallic zirconium, Zr (45 μm, purity 99.2 wt%, GF85844740 Aldrich, St. Louis, MO, USA), copper, Cu (75 μm, 99.99 wt%, 207780 Sigma–Aldrich, St. Louis, MO, USA), and tungsten, W (10 μm, 99.99 wt%, 357,421 Sigma–Aldrich, St. Louis, MO, USA) were used as the starting materials. The powders were balanced and then mixed inside He-atmosphere (99.99%) glove box (UNILAB Pro Glove Box Workstation, mBRAUN, Germany) to obtain an amount of 25 g with nominal composition of (Zr_67_Cu_33_)_100−*x*_W*_x_* (*x*; 0, 5, 10, 20, and 30 at%). Table 1 presents the detailed ICP analysis of the starting composition, given at% and wt%.

### 3.2. Cold Rolling and High-Energy Ball Milling Procedures

The mixed powders of each composition were charged into individual tool steel vials (500 mL in volume) and well-sealed together and with 75 tool steel balls (11 mm in diameter) in the glove box. The ball-to-powder weight ratio used was 20:1. The vials were then mounted on high-energy ball mill (Planetary Mill PULVERISETTE 5, Fritsch, Germany), where the BM process was carried out for 3 h at ambient temperature. The as-BM powders of each composition were discharged in the glove box and then sealed into five individual stainless steel tubes (1.0 cm diameter and 30 cm length). The tubes were then severely cold rolled (CR) for 30, 50, and 100 continuous passes. Few amount (3 g) of the CR powders obtained after each set of passes were discharged from the host-tube and kept in the glove box for different analysis. All of the powders with different composition that had been successfully CR for 100 passes were continuously high-energy ball milled for 36 h and 75 h.

### 3.3. Powder Consolidation by Spark Plasma Sintering (SPS)

**Figure 11:**
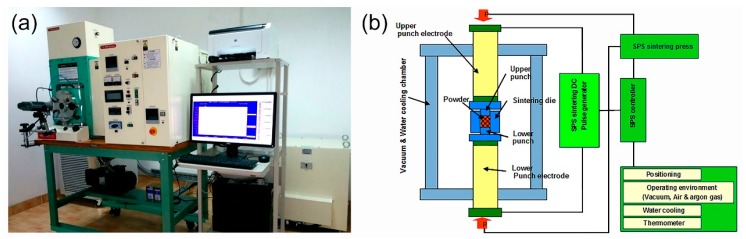
(**a**) A complete SPS system produced by Dr. Sinter Lab. Instrument, Japan housed in the Nanotechnology Laboratory, Energy and Building Research Center, Kuwait Institute for Scientific Research, and (**b**) schematic illustration of the system configuration.

**Figure 12:**
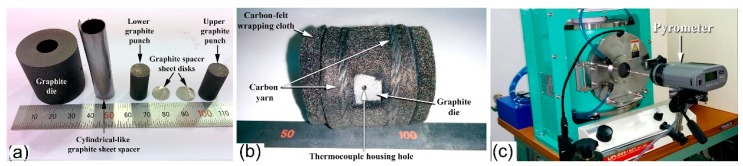
(**a**) Powder loading kits used in SPS consisting of graphite die and punches, cylindrical graphite spacer and graphite disk spacer, (**b**) the die containing the powder was wrapped by carbon-felt and tight with using carbon yarn, and (**c**) the surface temperature of the die was monitored during the SPS process, using a pyrometer.

The powders that were obtained after CR for 100 passes followed by 75 h of BM were individually consolidated into dense buttons, while using the SPS technique. A photo the SPS system (Dr. Sinter Lab. Instrument, Japan) and its configurations, used for the sintering process, are presented together in Figure 11a,b, respectively. The system consists of a sintering press unit with a vertical single-axis pressurization, special designed punch electrodes incorporating a water cooler, a water-cooled vacuum chamber, a vacuum/air/argon-gas atmosphere control mechanism, a special (direct current) DC pulse sintering power generator, a cooling-water control unit, *Z*-axis position measuring and control unit, temperature measuring and control units, an applied pressure display unit, and various safety interlock devices. In the present work, the as-BM powders were charged into a graphite die and then stacked between upper and lower punches (Figure 12a). Graphite sheets are used as spacers to ensure an easy ejection of the sample after sintering and in order to avoid any reactions between the internal surfaces of the die tools (die and punches) with the powders (sample) (see Figure 12a).

The entire die and punch assembly were wrapped with carbon felt, which is held closed using carbon yarn, for reducing the amount of radiant heat transfer to the machine, as shown in Figure 12b. The die was then mounted on the sintering stage in the SPS chamber and held between the upper punch and lower punch electrodes (Figure 11b). During the SPS process, temperature was set below the crystallization temperature of the metallic glassy powders of each composition. A pyrometer was used to measure the temperature of the die surface during sintering process, as displayed in Figure 12c.

In the present work, the powder sintering with SPS process was conducted by the presence of an electric field, known as the field assisted sintering technique (FAST). In contrast with the conventional sintering techniques in which the sample is heated from the outside, the sintering procedure in SPS was taken place upon internally heating the sample by the passage of an electric current, with extremely high heating and cooling rates of 580 and 280 K per min., respectively. The external pressures applied during the sintering process was in the range between 10–15 MPa. The whole process, including temperature ramp and holding times, took about 6 min.

### 3.4. Sample Characterizations

#### 3.4.1. Crystal Structure

The crystal structures of all samples were investigated by X-ray diffraction (XRD) with CuKα radiation, using 9kW Intelligent X-ray diffraction system, provided by SmartLab-Rigaku, Japan. The local structure of the synthesized materials was studied by 200 kV-field emission high resolution transmission electron microscopy/scanning transmission electron microscopy that was (HRTEM/STEM) supplied by JEOL-2100F, JEOL, Japan, and equipped with Energy-dispersive X-ray spectroscopy (EDS) supplied by Oxford Instruments, Oxford, UK. Cryo Ion Slicer Machine (IB-09060CIS) supplied by JEOL-2100F, JEOL, Japan was used to prepare bulk TEM samples of as-SPS buttons.

#### 3.4.2. Morphology and Elemental Analysis

The morphological characteristics of the milled and consolidated samples were investigated by means of field-emission scanning electron microscope (FE-SEM), while using 15 kV- JSM-7800F, JEOL-Japan. The local elemental analysis was investigated by the energy-dispersive X-ray spectroscopy (EDS, Oxford Instruments, Oxford, UK) system interfaced with the FE-SEM.

#### 3.4.3. Thermal Stability

Differential scanning calorimeter (DSC), which was provided by Setaram, France, using a heating rate of 40 °C/min, was employed to investigate the glass transition temperature, glass forming ability, and thermal stability indexed by the supercooled liquid region and crystallization temperature of the metallic glassy samples.

#### 3.4.4. Density and Vickers Microhardness

The density of the consolidated samples was measured by the Archimedean approach, while using toluene. The microhardness of the compacted sample was determined using a Vickers indenter with a load of 1 kg. The hardness values reported subsequently are averaged from at least 10 indentations.

## 4. Conclusions

Although it is impossible to prepare metallic glassy (Zr_67_Cu_33_)_100−*x*_W*_x_* alloys through the rapid solidification approach, the present study has shown the possibility of employing cold rolling accompanied with mechanical alloying approach to fabricate such new metallic glassy alloys with alloys. Based on the results of this study, the following conclusions can be driven:

(1) The system can be obtained successfully in wide W concentrations, extended from 5 to 30 at%.
(2) Pretreatment of the feedstock Zr, Cu, and W metal powders, using the cold rolling method, led to obtaining well-aligned multilayered structure particles. Increasing the cold rolling time to 100 passes enhanced the solid-state diffusion between Zr/Cu/W layered and led to obtaining a supersaturated solid-solution phase.
(3) When the solid solution powders were subjected to high-energy ball milling for 75 h, the bcc-solid solution phase could not withstand the severe plastic deformation and imperfections generated by the balls milling media and transformed into a metallic glassy phase.
(4) The as-fabricated (Zr_67_Cu_33_)_100−*x*_W*_x_* metallic glassy alloys revealed excellent GFA and good thermal stability, as indicated by their wide ∆T_x_, and high T_x_ values.
(5) Based on the their wide ∆T_x_ before crystallizations and high T_x_, the as-fabricated powders were consolidated into BMG buttons, while using SPS technique.
(6) The SPS consolidation step maintained the original short-range order structure after consolidation without experiencing any partial crystallizations.
(7) The metallic glassy consolidated buttons were nearly full dense (above 99.95%).
(8) The Vickers microhardness have shown a monotonical increase (from 2.8 ± 0.23 GPa to 9.7 ± 0.71 GPa Hv), depending on the W contents (5 at% to 30 at%).

## Figures and Tables

**Table 1:** Nominal and real compositions of as prepared bulk metallic glasses (BMG) (Zr_67_Cu_33_)_100−*x*_W_*x*_ systems.

Alloy (SN#)	1	2	3	4	5
Nominal Composition (at%)					
Zr	67	63.65	60.3	53.6	46.9
Cu	33	31.35	29.7	36.4	23.1
W	0	5	10	20	30
Nominal Composition (wt%)					
Zr	74.45	66.90	59.38	48.04	38.06
Cu	25.55	22.57	20.68	16.11	12.98
W	0	10.53	19.94	35.85	48.96
Real Composition after Processing and Consolidations (wt%)					
Zr	74.38	67.05	59.31	48.11	37.92
Cu	25.62	22.51	20.62	16.14	12.88
W	0	10.44	20.25	35.75	49.20
Fe-Contamination (that come from the balls) and Oxygen Contents (wt%)					
Fe	0.08	0.22	0.36	0.45	0.57
Oxygen	0.16	0.13	0.26	0.22	0.18

**Table 2:** EDS elemental analysis of the samples obtained after (a) 100 passes of CR *, and (b) 100 passes of CR, followed by BM for 75 h **.

Alloying Elements (wt%)				
Zone	Zr	Cu	W	Total
**(a) CR for 100 Passes**				
I	37.96	13.08	48.96	100
II	38.06	12.93	49.01	100
III	37.94	12.89	49.17	100
IV	38.09	13.02	48.89	100
V	37.91	13.12	48.97	100
VI	38.05	12.87	49.08	100
VII	38.08	12.99	48.90	100
VIII	37.98	13.02	49.00	100
IX	38.05	12.93	49.02	100
X	38.08	13.04	48.88	100
XI	37.97	12.91	49.12	100
XII	38.09	13.01	48.90	100
**(b) CR for 100 Passes + BM for 75 h**				
I	38.02	12.93	49.05	100
II	38.07	12.91	49.02	100
III	37.98	12.99	49.03	100
IV	38.08	12.95	48.97	100
V	38.07	12.97	48.96	100
VI	38.01	12.96	49.03	100
VII	37.97	13.06	48.97	100
VIII	37.95	13.08	48.97	100
IX	38.09	12.89	49.02	100
X	37.98	13.01	49.01	100
XI	38.09	13.03	48.88	100
XII	37.99	13.03	48.98	100

* Figure 3a. ** Figure 3b.
